# Spanish trauma icu registry (RETRAUCI). final results of the pilot phase

**DOI:** 10.1186/2197-425X-3-S1-A377

**Published:** 2015-10-01

**Authors:** M Chico-Fernández, JA Llompart-Pou, F Alberdi-Odriozolo, F Guerrero-López, M Sánchez-Casado, MD Mayor-García, J Egea-Guerrero, JF Fernández-Ortega, A Bueno-González, J González-Robledo, L Servià-Goixart, J Roldán-Ramírez, MÁ Ballesteros-Sanz, E Tejerina-Álvarez, GT Trauma y Neurointensivismo SEMICYUC

**Affiliations:** Hospital Universitario 12 Octubre, Madrid, Spain; Hospital Universitari Son Espases, Palma de Mallorca, Spain; Hospital Universitario de Donostia, San Sebastian, Spain; Hospital Universitario Virgen de las Nieves, Granada, Spain; Hospital Virgen de la Salud, Toledo, Spain; Complejo Hospitalario de Torrecárdenas, Almería, Spain; Hospital Universitario Virgen del Rocío, Sevilla, Spain; Hospital Universitario Carlos Haya, Málaga, Spain; Hospital General Universitario de Ciudad Real, Ciudad Real, Spain; Complejo Asistencial Universitario de Salamanca, Salamanca, Spain; Hospital Universitari Arnau de Vilanova., Lleida, Spain; Complejo Hospitalario de Pamplona, Pamplona, Spain; Hospital Universitario Marqués de Valdecilla, Santander, Spain; Hospital Universitario de Getafe, Madrid, Spain

## Introduction

Trauma registries are essential to understand the health care reality and underscore potential areas of improvement in trauma patient management.

## Objectives

To present a real picture of the epidemiology of severe trauma and its related attention in Spanish intensive care units (ICUs) through the final results of the pilot phase of the Spanish trauma ICU registry (RETRAUCI).

## Methods

Prospective multicenter registry of patients with trauma admitted in 13 Spanish ICUs. We evaluated epidemiology, out-of-hospital attention, registry of injuries, resources utilization, complications and outcome.

## Results

We evaluated 2242 patients. Mean age 47.1 ± 19.02 years. Male 79%. Blunt trauma 93.9%. Injury Severity Score 22.2 ± 12.1, Revised Trauma Score 6.7 ± 1.6. Non-intentional in 84.4%, most common causes for trauma were road traffic accidents followed by pedestrian and high-energy falls. Up to 12.4% were taking antiplatelets or anticoagulants. Close to 28% had suspected or confirmed toxic influence in trauma. Up to 31.5% required out-of-hospital artificial airway. Time between trauma and ICU admission was 4.7 ± 5.3 hours. At ICU admission, 68.5% remained hemodinamically stable. Twenty-six percent received blood transfusion within 6 hours of ICU admission. Brain and chest injuries were predominant. Complications occurred frequently: trauma-induced coagulopathy in 32.1%, rhabdomyolysis 11.1%, early and late MOF 10.9% and 15.7% respectively, ARDS 23.4%, renal failure 14.7% and nosocomial infection 32.3%. Intracranial pressure was monitored invasively in 21%. Of them 65.8% presented intracranial hypertension. Mechanical ventilation was used in 69.5% of the patients (mean 8.2 ± 9.9 days), of which 24.9% finally required a tracheostomy ICU and hospital length of stay were 10.1 ± 12.8 and 16.0 ± 20.8 days respectively. ICU mortality was UCI 12.3% (273 patients). In-hospital after ICU mortality was 3.7%. Of note, up to 11.6% were transferred to another ICU.

## Conclusions

The pilot phase of the RETRAUCI shows a real and precise picture of the epidemiology and attention of severe trauma patients admitted in Spanish ICUs.

## Grant Acknowledgment

Fundación Mutua Madrileña
